# Strain-Rate-Dependent Tensile Response of Ti–5Al–2.5Sn Alloy

**DOI:** 10.3390/ma12040659

**Published:** 2019-02-22

**Authors:** Bin Zhang, Jin Wang, Yang Wang, Yu Wang, Ziran Li

**Affiliations:** CAS Key Laboratory of Mechanical Behavior and Design of Materials, Department of Modern Mechanics, University of Science and Technology of China, Hefei 230027, China; zhb1005@mail.ustc.edu.cn (B.Z.); wj1995@mail.ustc.edu.cn (J.W.); wyu@ustc.edu.cn (Y.W.); lzr@ustc.edu.cn (Z.L.)

**Keywords:** Ti–5Al–2.5Sn alloy, strain rate, tension deformation and fracture, constitutive model

## Abstract

This study is an experimental investigation on the tensile responses of Ti–5Al–2.5Sn alloy over a wide range of strain rates. Uniaxial tension tests within the rate range of 10^−3^–10^1^ s^−1^ are performed using a hydraulic driven MTS810 machine and a moderate strain-rate testing system. The high-rate uniaxial tension and tension recovery tests are conducted using a split-Hopkinson tension bar to obtain the adiabatic and isothermal stress–strain responses of the alloy under dynamic loading conditions. The experimental results show that the value of the initial yield stress increases with the increasing strain rate, while the strain rate sensitivity is greater at high strain rates. The isothermal strain-hardening behavior changes little with the strain rate, and the adiabatic temperature rise is the main reason for the reduction of the strain-hardening rate during high strain-rate tension. The electron backscatter diffraction (EBSD) analysis of the post-deformed samples indicates that there are deformation twins under quasi-static and high-rate tensile loadings. Scanning electron microscope (SEM) micrographs of the fracture surfaces of the post-deformed samples show dimple-like features. The Zerilli–Armstrong model is modified to incorporate the thermal-softening effect of the adiabatic temperature rise at high strain rates and describe the tension responses of Ti–5Al–2.5Sn alloy over strain rates from quasi-static to 1050 s^−1^.

## 1. Introduction

Due to its high specific strength and excellent corrosion resistance, Ti–5Al–2.5Sn alloy has been employed in engineering applications such as aerospace and warship structures. Specifically, Ti–5Al–2.5Sn alloy is suitable for use in jet and steam turbine blades, as well as turbopumps, where high-rate loadings such as blasts, foreign object impact, and high-speed machining often occur [1,2,3]. Therefore, it is of great significance to understand and model the mechanical behavior of Ti–5Al–2.5Sn alloy at high strain rates for its structural applications, numerical simulations, and manufacturing processes [4,5].

For α-titanium alloys, experimental investigations of the stress–strain response in low strain-rate tension and high strain-rate compression have been reported. Previous studies have indicated that the mechanical response of Ti–5Al–2.5Sn alloy is sensitive to the strain rate and temperature [6,7,8,9,10]. It has been found that the flow stress of Ti–5Al–2.5Sn alloy increases with the increase of the strain rate and that the strain rate sensitivity parameter, determined from the slope of the log (flow stress) versus log (strain rate) curve, was about 0.3 under quasi-static loading conditions (10^−4^–10^−2^ s^−1^). The grain size, cavitation, and dynamic recrystallization also varies with the strain rate. Dynamic strain aging (DSA), characterized as serrations in the stress–strain curves, occur at a certain range of temperatures and strain rates [6,7]. Experimental results of the tension and tension creep tests of Ti–5Al–2.5Sn alloy at various testing temperatures have shown that basal and prismatic slip systems dominate tensile deformation, while grain boundary sliding predominates in creep deformation [8,9]. The high-rate compression response of Ti–5Al–2.5Sn alloy indicates that its plastic deformation behavior is sensitive to the strain rate, and there exists a significant strain-hardening effect in compression deformation [10]. Tensile tests of Ti–5Al–2.5Sn alloy have shown that the main fracture mode of this alloy is ductile fracture. The fracture surface presents as a dull and matte appearance. All fracture surfaces are dominated by dimple rupture [11,12].

Due to the wide application of titanium alloys in the aerospace industry, where high-rate loadings are common, there are many investigations on the constitutive relationships of titanium alloys at high strain rates [13,14,15,16,17]. To account for grain-size effect, the classical Johnson–Cook constitutive model was modified by adding a grain strain term to characterize the mechanical properties of Ti–6Al–4V alloy of different grain sizes over a wide range of strain rates. The prediction of the modified model is more accurate than that of the classical Johnson–Cook model [13]. The thermal softening term, strain-rate hardening term, and strain hardening term were multiplied to characterize the elevated temperature, high strain rate, and large plastic strain response of Ti–6Al–4V [14]. A radial basis function artificial neural network model was established to characterize the flow stress of Ti−13Nb−13Zr alloy at high strain rates, which exhibited excellent predictability [15]. A physically based model was proposed to describe the strength and flow stress of Ti–6Al–4V alloy, in which the phase chemical compositions, phase volume fractions, grain size, and grain morphology were taken into consideration [17].

It has been well established that α-titanium and its alloys exhibit tension–compression asymmetry as the result of the low symmetry of the hexagonal close-packed (HCP) structure [18,19]. However, few studies on the tension behavior of α-titanium alloy subjected to moderate strain-rate and high strain-rate loadings were documented due to technical difficulties. To evaluate the effect of strain rate on the tension properties of Ti–5Al–2.5Sn alloy for engineering applications, this article presents a systematical investigation on the tension behavior of Ti–5Al–2.5Sn alloy and the corresponding deformation and fracture pattern over wide strain rates from 10^−3^ to 10^3^ s^−1^. Furthermore, the modified physically based Zerilli–Armstrong constitutive model is used to characterize the rate-dependent tension behavior of Ti–5Al–2.5Sn alloy.

## 2. Materials and Methods

**Figure 1:**
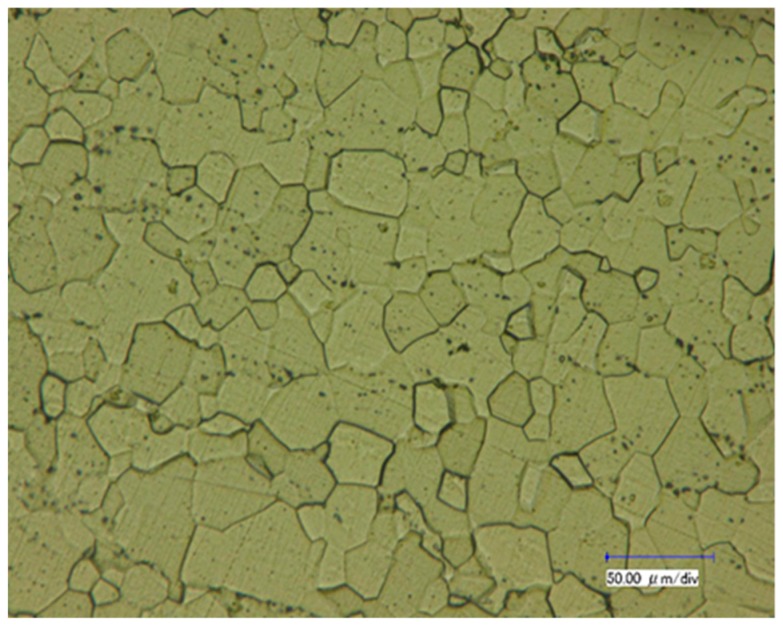
The microstructure of the annealed Ti–5Al–2.5Sn rod.

The material investigated in this work was a polycrystalline α-titanium forged and rolled rod. The chemical composition (wt.%) was reported in Table 1. The as-received condition rod was annealed at 800 °C for 3 h and then cooled down to room temperature. The microstructure of the annealed alloy presents as equiaxial grains, and only the α phase exists, as shown in Figure 1. It should be pointed out that the dark morphologies between some grains result from excessive etching due to the low activities of some grain boundaries.

To quantify the effect of the strain rate on the stress–strain response of Ti–5Al–2.5Sn alloy in uniaxial tension, a combination of quasi-static, moderate strain rate, and dynamic testing techniques was adopted. The strain rate tested in this study ranged from 10^−3^ to 10^3^ s^−1^. The quasi-static tension test was performed on a MTS810 servo-hydraulic machine (MTS systems corporation, Eden Prairie, MN, USA) equipped with a 100 kN capacity force transducer. An extensometer measured the strains with a gauge length of 25 mm. A constant crosshead speed mode realized the tension procedure. The test at moderate strain rates ranging from 1 to 10 s^−1^ was conducted on a moderate strain-rate testing apparatus. This hydraulically driven testing apparatus consisted of an inner impact module to achieve the destined loading velocity, and a buffer to reduce the oscillation brought on by the impact. The loading velocity was adjusted by changing the velocity of the piston rod driven by the hydraulic power unit. The load applied to the tensile sample and the axial strain of the sample was measured by a force transducer and an optical extensometer, respectively. A detailed description of the testing set-up and measurement technique can be found elsewhere [20]. A high-rate tension test was conducted on a modified split-Hopkinson tension bar system [21,22]. Based on the incident and transmitted strain histories of the bars measured by the strain gages, the stress and strain rate of the sample was obtained. In particular, a prefixed short metal bar made of approximate, elastic, perfectly plastic aluminum was used in the present work to generate the smooth incident tensile loading pulse. The rise time, duration, and amplitude of the tensile pulse were determined by the impact velocity and the diameter and length of the prefixed metal bar, which means that the strain rate could be altered easily. The dumbbell-shaped flat sample was sandwiched between the incident and transmitted bars with a high-strength adhesive. The pseudo constitutive response was eliminated as the result of the relatively smooth and stable incident/transmitted waves and the reliable sample/bars connection.

The dimension of the dumbbell-shaped flat sample used in the high-rate test was chosen to be 10 mm gauge length, 3.5 mm width, 2 mm radius fillet, and 1.1 mm thickness [23]. The sample geometry used in the quasi-static test was similar, except that the gage length and radius fillet were 25 mm and 20 mm, respectively, to avoid the end effects. The gauge length and width of the sample used in the moderate strain-rate test were 10 mm and 7 mm, respectively. All the samples were prepared from the same annealed rod by wire electro-discharge machining. The tensile loading direction was parallel to the axial direction of the rod. At least three tests were conducted at each strain rate to ensure that the obtained stress–strain curves coincided well.

The post-mortem was conducted on the tension samples deformed at the two extreme strain rates, i.e., 0.001 s^−1^ and 1050 s^−1^. Electron backscattered diffraction (EBSD) observation was performed to obtain the microstructural orientation information of the alloy after tension loading. The post-deformed samples were first mechanically polished, and then, ion etching was performed for precision polishing. The fracture surfaces of the tension samples were examined by a scanning electron microscope (SEM) to determine the fracture mode.

## 3. Results and Discussion

**Figure 2:**
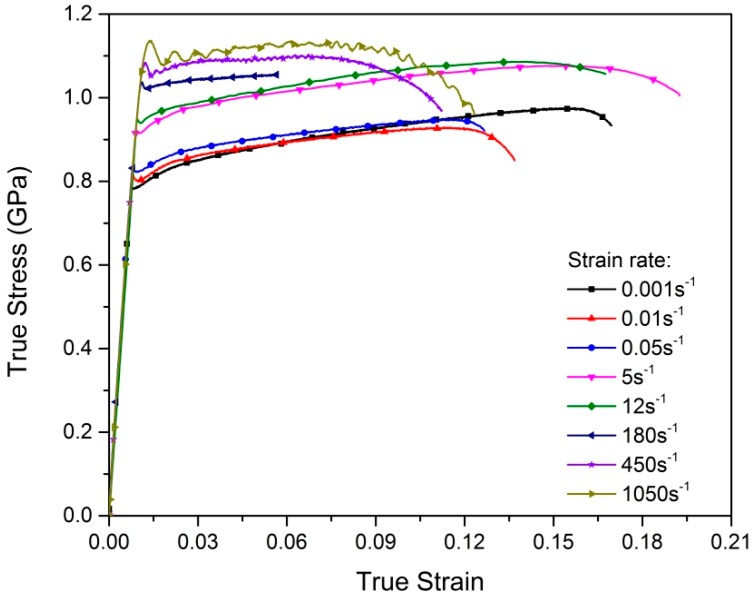
Experimental stress–strain responses in uniaxial tension for Ti–5Al–2.5Sn alloy.

Figure 2 shows the true stress–true strain responses of Ti–5Al–2.5Sn alloy in uniaxial tension at room temperature and eight strain rates from 0.001 to 1050 s^−1^. The obtained stress–strain curves indicate that the tension response of Ti–5Al–2.5Sn alloy has noticeable strain-rate-dependent plastic deformation characteristics. There was an increase in the initial yield stress with the increasing strain rate, which demonstrates the positive strain-rate dependence. However, the strain-hardening rate decreased at high strain rates. It is worth noting that this decline did not exhibit a strict monotonic trend. There was a visible decreasing in the strain-hardening rate when the strain rate was increased from 0.001 s^−1^ to 0.01 s^−1^. Nevertheless, the strain-hardening rate changed little when the strain rate was increased up to 12 s^−1^. A sudden decreasing of the strain hardening occurred at the rate of 180 s^−1^. Furthermore, the strain hardening changed minimally in the rate range from 180 s^−1^ to 1050 s^−1^ corresponding to dynamic loadings. It is important to note that the plastic work is converted into heat during plastic deformation, and the heat loss due to heat conduction and radiation depends on the level of strain rate. A large portion of this heat remains inside a sample subjected to high-rate loadings, which leads to thermal softening in the stress–strain response, and the plastic deformation at high strain rates is essentially adiabatic [24]. The mechanical behavior of the alloy at high strain rates is the coupling of the strain-rate strengthening, strain hardening, and thermal softening. Hence, the decrease of the strain-hardening rate of the alloy at high strain rates of 180 s^−1^, 450 s^−1^, and 1050 s^−1^ solely comes from the adiabatic temperature-rise softening. In order to investigate the strain hardening and thermal softening separately, a recovery test for obtaining the high-rate isothermal stress–strain response is needed for the decoupling of the thermo-mechanical behavior of the alloy.

**Figure 3:**
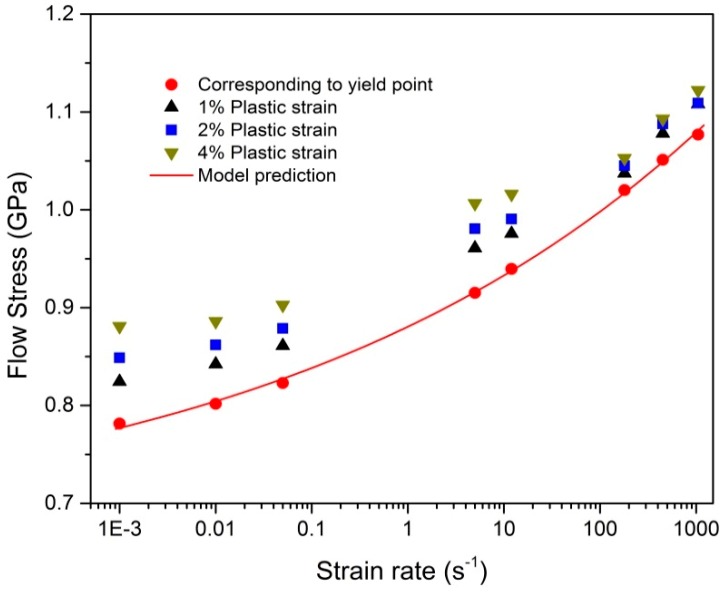
Flow stress versus logarithm strain rate.

The relation between flow stress and logarithm strain rate is shown in Figure 3. It is to be noted that the flow stress is sensitive to the strain rate and monotonically increases with the increase of the strain rate. At moderate and high rate range, flow stress is more sensitive to the strain rate than that at quasi-static rate range. The similar rate-sensitivity phenomenon was also observed for commercially pure Ti (CP Ti) in compression [25]. In order to quantify the effect of the strain rate on the initial yield stress, the strain-rate sensitivity (SRS) parameter of the alloy is defined as [26]

(1) $$\mathrm{SRS}=\frac{\Delta \sigma_{y}}{\Delta \log_{10}{\dot{\varepsilon }}_{p}}$$

where *σ*_*y*_ is the initial yield stress and ${\dot{\varepsilon }}_{p}$ is the axial plastic strain rate. The calculated SRS parameters of the alloy within the tested rate ranges are listed in Table 2. It is apparent that the value of the SRS parameter is much greater at moderate and high strain rates than that at low strain rates, and it increases almost monotonically with the increasing strain rate. The increase of the value of the SRS parameter is obvious in the quasi-static rate range of 0.01–0.05 s^−1^. When the strain rate exceeds 180 s^−1^, the values of the SRS parameter change. It can be concluded that there is a strain-rate sensitivity transition between 0.05 s^−1^ and 5 s^−1^. As described above, there also exists a strain-hardening rate transition between 12 s^−1^ and 180 s^−1^. Such phenomenon indicates that the moderate strain-rate test is necessary in order to more accurately analyze the rate-dependent tension behavior of Ti–5Al–2.5Sn alloy. As for the rate sensitivity of the flow stress at the plastic strains of 1%, 2%, and 4%, a similar rate-sensitivity transition phenomenon exists within the moderate rate range from 10^−1^ s^−1^ to 10^1^ s^−1^.

**Figure 4:**
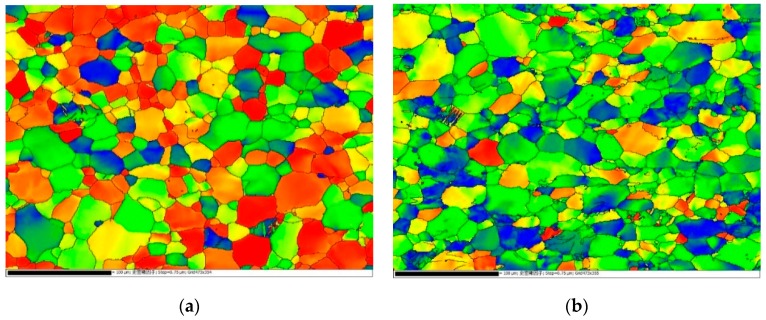
Electron backscatter diffraction (EBSD) maps of samples tested at (**a**) 0.001 s^−1^ and (**b**) 1050 s^−1^.

Figure 4 displays the microstructural orientation information through EBSD analysis of the tension samples deformed at two extreme strain rates investigated in the present paper. The EBSD maps were obtained from the area near the fracture surface in the gage section. Based on the characterization of the misorientation angles, the deformation twins in post-deformed samples were identified as $\left\{1\;0\;\bar{1}\;2\right\}$ twins, which had misorientation angles of 86°. However, the deformation twin density in the post-deformed samples was very low. Twinning was also rarely observed in tension and tension creep tests for Ti–5Al–2.5Sn alloy in previous works [8,9]. For CP Ti, because α-titanium is a hexagonal close-packed (HCP) structure with a c/a ratio of 1.587, deformation twinning occurs in CP Ti under both compression and tension loadings to maintain the deformation compatibility [25,27]. Previous investigations indicated that the deformation twinning may significantly increase the strain-hardening rate in CP Ti, and it is more likely to occur at high strain rates in CP Ti [25,27,28,29,30]. However, the addition of aluminum in Ti–5Al–2.5Sn alloy leads to an increase of the c/a ratio, resulting in the reduction of < c + a > activity and the suppression of twinning. The absence of deformation twinning means that slip predominates the deformation in Ti–5Al–2.5Sn alloy [31,32]. The low strain-hardening rate, as seen in Figure 2, may be due to the low deformation twinning density and the predomination of slip.

**Figure 5:**
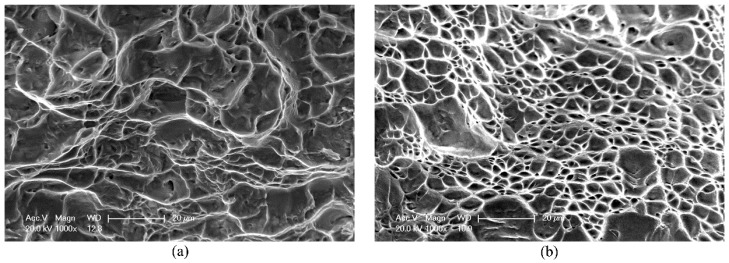
SEM observation of fracture surfaces for samples tested at (**a**) 0.001 s^−1^ and (**b**) 1050 s^−1^.

SEM observation was used to examine the fracture patterns of the samples deformed at different strain rates. Figure 5 shows the fracture surfaces of samples broken at 0.001 s^−1^ and 1050 s^−1^. The SEM images were obtained in the fibrous zones of the fracture surfaces. Cleavage facets and dimples can be seen under the quasi-static loading, and round dimples can be found at the high strain rate. The dimple rupture morphology observed in all the fractured samples indicates that Ti–5Al–2.5Sn alloy is broken in a ductile manner within the rate range investigated in this paper. Moreover, microvoid coalescence is the dominant fracture mode in all the tensile samples. It was reported that the Ti–5Al–2.5Sn extra-low-interstitial (ELI) alloy has excellent ductility, and the dimple morphology appears in the fracture surface when the testing temperature is as low as 8 K [2]. Also, a similar ductile fracture mechanism for Ti–5Al–2.5Sn alloy subjected to tension loadings can be found in the references [11,12]. Furthermore, it is seen in Figure 5 that the dimples become smaller at the rate of 1050 s^−1^. It is noted that the capability of plastic deformation for Ti–5Al–2.5Sn alloy subjected to high-rate loading decreases, as shown in Figure 2. Therefore, a positive correlation between dimple size and ductility for Ti–5Al–2.5Sn alloy exists in the present investigation.

**Figure 6:**
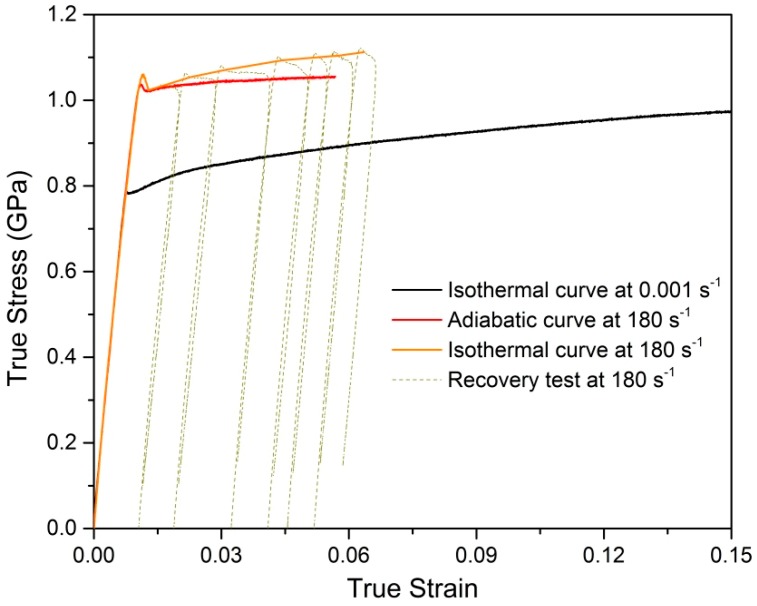
Comparison of isothermal and adiabatic responses.

As described above, the strain hardening, strain-rate strengthening, and adiabatic temperature-rise softening simultaneously affect the plastic deformation behavior of Ti–5Al–2.5Sn alloy, as well as cause them to couple to each other during dynamic loadings. In order to achieve the thermo-mechanical decoupling in dynamic tension, a dynamic recovery experiment was carried out to obtain the isothermal stress–strain curve at the rate of 180 s^−1^ [33,34,35]. The comparison between the adiabatic stress–strain curve at 180 s^−1^ and the isothermal curves at 180 s^−1^ is shown in Figure 6. The strain-hardening rate in the adiabatic tension deformation is much lower than that in the isothermal tension deformation, which is solely attributed to the adiabatic temperature rise. The loading process, at the rate of 0.001 s^−1^, can be considered to be isothermal due to the extremely slow loading speed. Due to the absence of adiabatic softening, the difference between the isothermal tension curves of 0.001 s^−1^ and 180 s^−1^ only comes from the strain rate that the sample experienced. It is observed that the isothermal tension curves obtained at 0.001 s^−1^ and 180 s^−1^ are almost parallel, which indicates that the isothermal strain-hardening behavior of the material is almost the same and independent of the strain rate. In other words, the strain rate only strengthens initial yield stress and has little effect on strain hardening of Ti–5Al–2.5Sn alloy in the tested strain-rate range.

A dislocation-mechanics-based constitutive relation has been proposed by Zerilli and Armstrong [36]. For materials with body-centered cubic (BCC) structure, the flow stress in the Zerilli and Armstrong constitutive relation (ZA model) can be given as functions of plastic strain, *ε*, strain rate, $\dot{\varepsilon }$, and temperature, *T*.

(2) $$\sigma =\sigma_{a}+B_{1}e^{-\left(\beta_{1}-\beta_{2}ln\dot{\varepsilon }\right)T}+B_{2}\varepsilon^{n}$$

where *σ_a_*, *B*_1_, *β*_1_, *β*_2_, and *B*_2_ are the model constants. The first and the second terms in the relation indicate that the initial yield stress is associated with the intrinsic properties of the metal and the loading conditions including both temperature and the strain rate, respectively. The third term represents the strain-hardening behavior. For Ti–5Al–2.5Sn alloy, its initial yield stress increases with the increase of the strain rate, and this increasing trend is nonlinear with the logarithm of the strain rate within the rate range investigated in this paper. Such a trend may be characterized by the exponential function, which is expressed in the ZA model. As for the strain-hardening behavior of Ti–5Al–2.5Sn alloy, the isothermal strain-hardening rate is independent of the strain rate, as shown in Figure 6, which is consistent with the expression in the ZA model, because there is no strain-rate related parameter introduced in the strain-hardening term. Thus, the ZA constitutive model was adopted to characterize the tension responses of Ti–5Al–2.5Sn alloy at various strain rates. Nevertheless, this constitutive model does not consider the adiabatic softening effect under high-rate loadings. In order to accurately describe the tension behavior of the alloy at high strain rates, the effect of adiabatic softening should be added to the strain-hardening term in the ZA model. Because all experiments in this study were conducted at room temperature, it is not necessary to take the effect of testing temperature into account in the constitutive model. In summary, the constitutive model can be modified as

(3) $$\sigma =\sigma_{a}+B_{1}e^{\beta ln\dot{\varepsilon }}+B_{2}\varepsilon^{n}e^{-\lambda \Delta T}$$

where *e*^−*λ*Δ*T*^ in the third term denotes the effect of adiabatic softening on strain hardening, *λ* is the thermal softening coefficient [37], and Δ*T* is the adiabatic temperature rise, which can be calculated by

(4) $$\Delta T=\frac{\eta }{\rho C_{V}}\int \sigma d\varepsilon$$

where *ρ* is the mass density, *C_v_* is the specific heat capacity, and *η* is the conversion coefficient of plastic work to heat.

**Figure 7:**
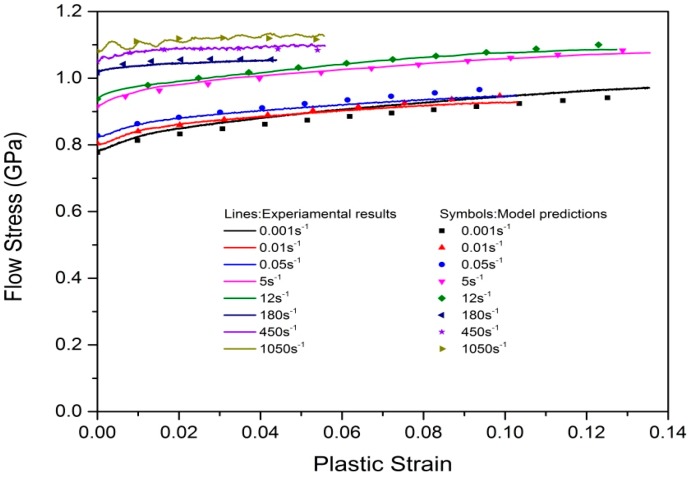
Comparison of the experimental results and model predictions.

As for the evaluation of the model constants, *σ_a_*, *B*_1_, and *β*_1_ can be obtained by fitting the variation of the initial yield stress with the strain rate in Figure 3. The good agreement shown in Figure 3 indicates that this constitutive model accurately characterizes the rate dependence of the initial yield stress. *B*_2_ and *n* can be obtained by fitting isothermal stress–strain curves at 0.001 s^−1^, 0.01 s^−1^, 0.05 s^−1^, and 180 s^−1^. The adiabatic softening coefficient *λ* can be derived by comparing the isothermal and adiabatic tension curves at 180 s^−1^ as follows:

(5) $$\lambda =\frac{1}{\Delta T}ln\frac{B_{2}\varepsilon^{n}}{\sigma_{adiabatic}-\sigma_{isothermal}+B_{2}\varepsilon^{n}}$$

where $\sigma_{adiabatic}$ and $\sigma_{isothermal}$ correspond to the flow stresses obtained from the adiabatic and isothermal experimental results at high strain rates, respectively. For Ti–5Al–2.5Sn alloy, *ρ* = 4.48 × 10^3^ Kg/m^3^ and *C_v_* = 530 J/(Kg·K) at room temperature. The conversion coefficient of plastic work to heat η is assumed to be 0.9 under dynamic loading conditions [34,37]. The plastic deformation processes at the strain rates lower than 180 s^−1^ were assumed to be under isothermal conditions. The final values of the model constants were determined by means of the least square method, as listed in Table 3. The material constants are of the same order of magnitude as the material constants obtained by other studies on CP Ti [38]. The comparison between experimental results and model results is presented in Figure 7. The description capability of the ZA models is assessed quantitatively by a model description error (MDE) defined as:

(6) $$\mathrm{MDE}=\frac{1}{n}\overset{n}{\underset{i}{\sum }}\left|\frac{\sigma_{exp}^{i}-\sigma_{model}^{i}}{\sigma_{exp}^{i}}\right|\times 100\%$$

where *σ_exp_* and *σ_model_* are the experimental result and model prediction, respectively [39]. The calculated values of MDE are 1.46%, 0.43%, and 0.63% corresponding to quasi-static, moderate-rate, and high-rate loading conditions, respectively. The overall average value of MDE is 0.89%. Such description error is extremely low compared with the results presented in the literature [39], which indicates that the modified ZA constitutive model can accurately characterize the tension responses of Ti–5Al–2.5Sn alloy in a wide range of strain rates. It is noted that the description error is as low as 0.63% at high strain rates, which means that the model results are in good agreement with the experimental stress–strain results at high strain rates, and the adiabatic temperature-rise softening term added in the model in the form of an exponential function is suitable for characterizing the adiabatic plastic deformation behavior.

## 4. Conclusions

Moderate strain-rate tension tests using the hydraulic-driven loading system and high-rate tension tests using the split-Hopkinson bar technique were conducted at room temperature to evaluate the strain-rate sensitivity of α-titanium Ti–5Al–2.5Sn alloy. The stress–strain curves in uniaxial tension in a rate range of 0.001–1050 s^−1^ were obtained. Based on the experimental data, it can be concluded that the investigated alloy shows rate-dependence in plastic behavior. The initial yield stress exhibits the positive strain-rate dependence, while the strain-hardening rate decreases at high strain rates, which results from the adiabatic temperature rise. The strain-rate sensitivity at high strain rates is more remarkable than that under quasi-static loadings. Deformation twins can be found in the post-deformed tension samples, and the twinning density does not change significantly with strain-rate. SEM observations of fracture surfaces indicate that the tensioned sample was broken in the ductile mode. High-rate tension recovery tests were carried out to obtain the isothermal stress-strain response of Ti–5Al–2.5Sn alloy subjected to high strain-rate loadings. The experimental results indicate that the strain rate strengthens the initial yield stress and has little effect on the isothermal strain-hardening rate. The adiabatic temperature rise is the main reason for the difference in the strain-hardening rate between adiabatic and isothermal loadings at high strain rates. The physically based Zerilli and Armstrong constitutive relation was modified to account for the adiabatic temperature-rise softening phenomenon in the plastic deformation at high strain rates, and the modified model was utilized to characterize the stress–strain response of Ti–5Al–2.5Sn alloy. A comparison between the model results and experimental data was made, and the good description capability of the modified constitutive relation was presented.

## Figures and Tables

**Table 1:** The chemical composition of the as-received Ti–5Al–2.5Sn.

Al	Sn	Fe	C	N	H	O	Ti
4.96	2.56	0.08	0.013	0.011	0.001	0.14	balance

**Table 2:** Strain rate sensitivity (SRS) within various strain-rate ranges.

Rate Range (s^−1^)	0.001–0.01	0.01–0.05	0.05–5	5–12	12–180	180–450	450–1050
SRS (GPa)	0.0203	0.0304	0.0461	0.0644	0.0683	0.0776	0.0703

**Table 3:** Material constants of the modified Zerilli and Armstrong constitutive relation constitutive model (ZA).

*σ*_a_ (GPa)	*B*_1_ (GPa)	*β*	*B*_2_ (GPa)	*N*	λ (K^−1^)
0.665	0.216	0.094	0.566	0.595	0.048
